# Redefining and solving the digital divide and exclusion to improve healthcare: going beyond access to include availability, adequacy, acceptability, and affordability

**DOI:** 10.3389/fdgth.2025.1508686

**Published:** 2025-04-22

**Authors:** Laronda A. Hollimon, Kayla V. Taylor, Rachel Fiegenbaum, Mary Carrasco, Laurent Garchitorena Gomez, Debbie Chung, Azizi A. Seixas

**Affiliations:** ^1^ The Media and Innovation Lab, Department of Informatics and Health Data Sciences, University of Miami Miller School of Medicine, Miami, FL, United States; ^2^ Department of Psychiatry and Behavioral Health Sciences, University of Miami Miller School of Medicine, Miami, FL, United States; ^3^ Frost Institute for Data Science and Computing, University of Miami, Miami, FL, United States

**Keywords:** access, affordability, broadband, internet, digital divide, health equity, digital health, equity

## Abstract

The digital divide in the United States extends beyond the traditional definition of access, which focuses solely on physical infrastructure like broadband networks and connectivity points. This narrow framing has resulted in policies that fail to address the full spectrum of barriers to digital inclusion. To bridge this gap, we propose the Rhizomatic Digital Ecosystem Framework, which emphasizes five interdependent components: access, availability, adequacy, acceptability and affordability. Access highlights the need for physical infrastructure, with programs like the Broadband Equity, Access, and Deployment (BEAD) Program expanding connectivity to underserved areas. Availability ensures the presence of reliable internet infrastructure to meet community needs, with targeted policies like satellite-based solutions addressing challenges in rural and Indigenous areas. Availability policies should focus on federal funding programs like BEAD and the Tribal Broadband Connectivity Program, incentivizing Internet service Providers (ISP) to expand into underserved areas, and leveraging satellite technologies to address infrastructure gaps. Novel policies to address the digital divide include community-owned broadband networks, dynamic spectrum sharing, and blockchain-powered micro-networks to improve availability in underserved areas. Adequacy examines whether internet services meet modern demands, such as telehealth and online education, emphasizing the need for minimum speed standards and performance improvements. Adequacy policies should include enforcing FCC speed standards with regular audits, requiring ISPs to upgrade outdated infrastructure, and providing government grants to improve broadband quality in communities. For adequacy, solutions like AI-driven broadband performance monitoring, funding edge computing in remote regions, and treating broadband as a public utility can enhance internet speed and quality Acceptability tackles cultural and social barriers, including digital literacy gaps, language differences, and technophobia, which can be addressed through localized literacy programs and inclusive design practices. Acceptability policies should prioritize national digital literacy campaigns for underserved groups, mandate inclusive design and accessibility standards, and offer multilingual and culturally relevant resources for digital tools. Affordability addresses financial barriers, as many low-income households struggle to afford monthly internet fees and devices, even with subsidies such as the Affordable Connectivity Program (ACP). To improve acceptability, innovative approaches like universal digital literacy vouchers, gamified education platforms, and mandatory accessibility standards for all digital technologies can ensure equitable and inclusive digital participation. Together, these five dimensions provide a nuanced and actionable framework for crafting effective, interconnected policies and solutions. By addressing each dimension through the lens of the Rhizomatic Digital Ecosystem Framework, policymakers can develop holistic strategies to eliminate the digital divide and foster equitable digital inclusion across all communities.

## Introduction

The National Digital Inclusion Alliance defines the digital divide as the disparity among individuals and between communities in their ability to access, and effectively engage with digital technologies due to differences in infrastructure, skills, and support. However, the digital divide extends beyond mere access. It is a multidimensional and systemic inequity driven by disparities in digital infrastructure, financial constraints, digital literacy, and the availability of culturally and linguistically inclusive digital content. Rooted in socioeconomic, geographic, racial, and policy-driven determinants, the digital divide disproportionately impacts marginalized communities, limiting their ability to engage in education, employment, healthcare, and civic participation. This persistent inequity exacerbates social stratification, widens economic disparities, and restricts opportunities for full participation in the digital economy and society (1–4).

The predominant framing of the digital divide has narrowly focused on access as the primary challenge, leading to policies that treat connectivity as a standalone solution while neglecting the broader structural inequities that shape digital participation. However, this narrow definition overlooks critical factors—such as affordability, adequacy, availability, and acceptability—that determine whether individuals can meaningfully engage with digital tools and fully participate in an increasingly digital society (5, 6). Access refers to the presence of foundational infrastructure enabling connectivity, in essence, acting as a bridge that will connect to the rest of main dimensions. Access alone does not guarantee meaningful engagement, as critical dimensions such as availability, adequacy, acceptability, and affordability also play significant roles in shaping digital participation. Availability pertains to the pre-existing physical infrastructure that is required for reliable internet, adequacy ensures that speeds meet modern demands, acceptability addresses social and cultural barriers to digital engagement, and affordability considers both the initial and ongoing costs of digital technologies. The neglect of these interconnected components has resulted in fragmented, low-impact, and unsustainable policy and interventions. Federal programs such as the Affordable Connectivity Program (ACP), Broadband Equity, Access, and Deployment (BEAD) Program, and Digital Equity Act (DEA) have provided targeted and unfortunately limited solutions, and thus their scope have been insufficient to comprehensively eliminate the digital divide. This paper argues for a redefinition of the digital divide to include these broader dimensions, advocating for a holistic framework that integrates. Combining access, availability, adequacy, acceptability, and affordability in our understanding of the digital divide can lead to more comprehensive understandings of causes and antecedents and thus effective, wholistic, and inclusive ameliorative policies to eliminate digital inequities and advance true digital inclusion.

Policymakers have long centered digital equity initiatives around affordability, assuming that reducing financial barriers would automatically lead to widespread access and inclusion. This assumption has driven programs such as the Affordable Connectivity Program (ACP), which provided a $30 monthly subsidy ($75 for Tribal lands) to help low-income households afford broadband services (7, 8). However, when ACP funding lapsed in May 2024, its shortcomings became evident—13% of recipients immediately canceled their internet service, and another 12% planned to do so (9, 10). Without sustained affordability interventions, more than half of former ACP participants now struggle to pay their internet bills, leading to service downgrades or disconnections. Additionally, the New York State broadband affordability mandate, which required ISPs to offer low-cost plans at $15 for 25 Mbps and $20 for 200 Mbps, triggered an unintended consequence—major providers withdrew their services, citing financial unsustainability (11). The failure of these affordability-driven solutions underscores a fundamental issue: affordability alone does not solve the digital divide. While reducing cost barriers is essential, it is insufficient without parallel efforts addressing availability, adequacy, and acceptability.

The limitations of affordability-based policies are further illustrated by fragmented and inconsistent broadband expansion efforts. While initiatives such as the Broadband Equity, Access, and Deployment (BEAD) Program, the Capital Projects Fund (CPF), and the Digital Equity Act (DEA) aim to improve digital access, their success is constrained by infrastructure gaps and inadequate broadband quality (12–14). For example, in rural and Indigenous communities, broadband infrastructure remains unreliable even with subsidies, rendering affordability measures ineffective. In Chattanooga, Tennessee, municipal investment in a city-owned fiber-optic network demonstrated a more sustainable solution by delivering high-speed internet to thousands of residents, reducing unemployment, and bridging gaps in digital education (15, 16); In New York City they are providing free or discounted internet services specifically to residents of the New York Public Housing Authority (NYCHA) through initiatives like Big Apple Connect (17). Yet, such public ownership models remain underutilized due to entrenched policy preferences favoring private-sector-driven affordability solutions. In healthcare, digital disparities persist despite affordability programs, as access to telehealth and electronic health records is often hindered by low broadband speeds, digital literacy barriers, and service inconsistencies (18–22). The COVID-19 pandemic magnified these inequities, yet policy responses remained short-term, failing to create systemic changes that integrate affordability with availability and adequacy (23).

To achieve true digital inclusion, policymakers must move beyond the narrow focus on affordability and adopt a multi-dimensional approach that incorporates infrastructure expansion, minimum service quality standards, and culturally tailored digital literacy programs. Programs must prioritize long-term investments in broadband infrastructure, incentivize ISPs to expand into underserved regions, and mandate performance-based accountability for service quality. Moreover, digital literacy initiatives must be redesigned to address accessibility and cultural barriers, particularly for linguistically diverse and historically marginalized communities (23–28). The failure of affordability-centric policies demonstrates the need for holistic strategies that view access as an intersection of affordability, availability, adequacy, and acceptability. Without comprehensive interventions, affordability measures will continue to offer temporary relief without addressing the structural inequities that perpetuate the digital divide.

## Rhizomatic digital ecosystem framework as a solution to the digital divide and exclusion in healthcare

Traditional approaches to the digital divide often rely on a linear relationship between access and affordability, assuming that lowering costs will inherently increase access. However, this view overlooks the interconnected nature of the digital divide, which also includes availability, adequacy, and acceptability. The *Rhizomatic Digital Technology Framework* provides a more comprehensive perspective, emphasizing the interconnectedness of these components.

**Figure 1:**
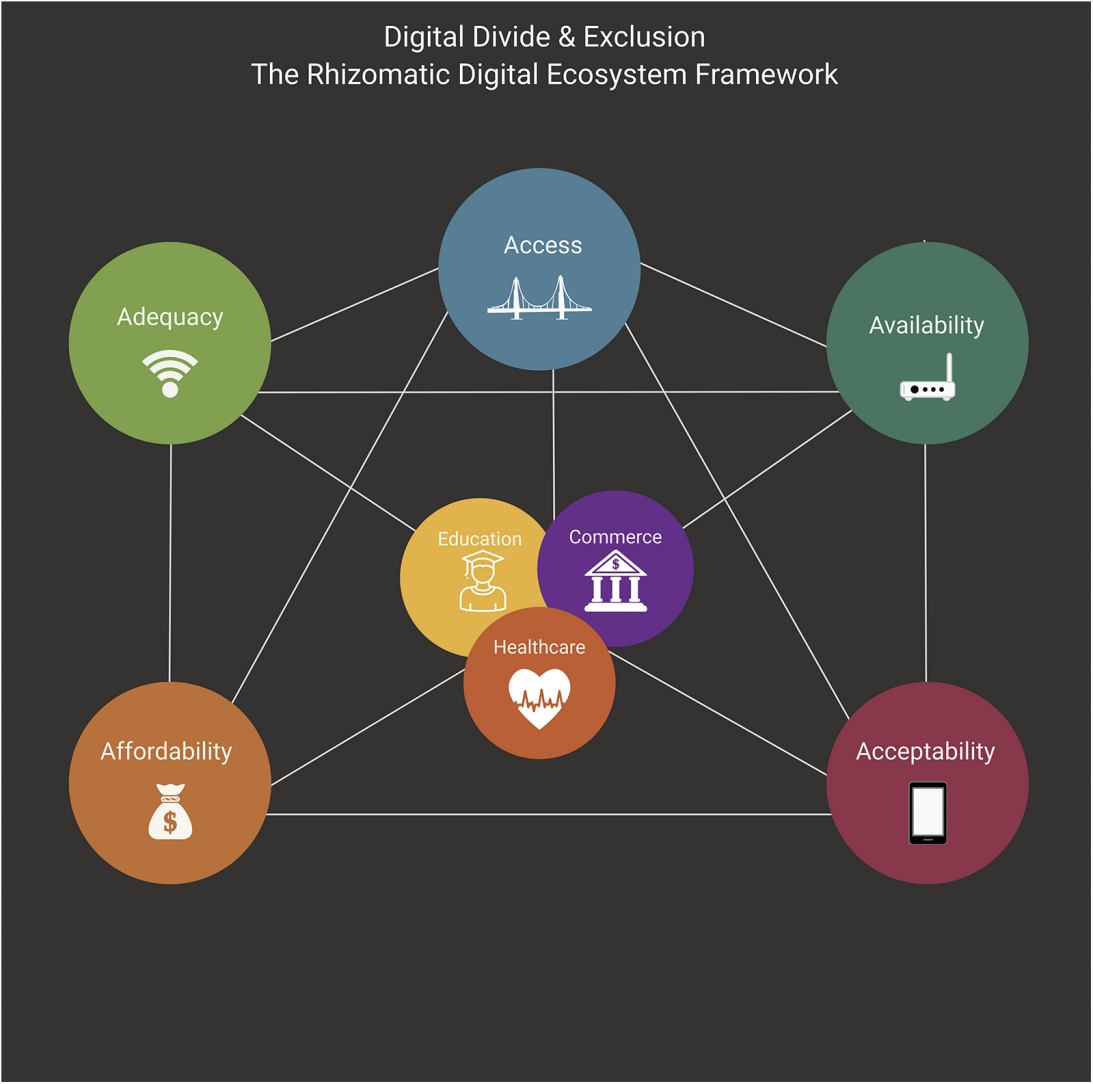
Components of digital divide and exclusion: deconstructing access to broadband internet and digital technologies. Access, Availability, Adequacy, Acceptability, and Affordability centers around healthcare, education, and commerce to represent key aspects of everyday life, illustrating how the digital divide impacts these essential areas. This highlights the central role of the internet and technology in shaping these experiences, emphasizing their essential impact on daily life. Digital divide and exclusion framework definition: Access—highlights the need for physical infrastructure, with programs like the broadband equity, access, and deployment (BEAD) program expanding connectivity to underserved areas. Availability—which focuses on the fundamental physical infrastructure—like fiber optic cables, cell towers, and broadband networks—that must exist for internet access to be possible, particularly challenging in rural and remote communities. Adequacy—examines whether the available internet service meets modern needs, as connection quality and speed can vary dramatically between locations and service providers. Acceptability—recognizes that even when technology is physically present and working well, social and cultural factors like language barriers, technological hesitation, or limited digital skills can prevent people from fully engaging with digital tools. Affordability—acknowledges that many individuals and families may be unable to pay for internet service, devices, and digital resources even when they are technically available and adequate for their needs.

To fully grasp the transformative potential of the *Rhizomatic Digital Ecosystem Framework*, it is crucial to unpack the concept of the rhizome and its application to technology and digital health. A rhizome, as described by Deleuze and Guattari, is a non-hierarchical, interconnected, and decentralized structure where every point is linked to every other, without a singular starting or end point (29). This contrasts with traditional, linear models that rely on top-down structures, where issues are often addressed in isolation. The rhizomatic framework rejects these linear hierarchies and instead emphasizes the dynamic, interconnected relationships between critical components of the digital ecosystem—access, availability, adequacy, acceptability, and affordability. In this way, it mirrors the realities of the digital world, where technology, infrastructure, and human engagement are deeply interwoven (Figure 1).

The rhizomatic framework introduces a transformative paradigm shift, urging us to view the digital ecosystem as a dynamic, interconnected system rather than a static set of challenges with linear solutions. This perspective emphasizes the interdependence of components—investments in infrastructure (access and availability) must coincide with efforts to ensure technology's relevance and usability (adequacy and acceptability), while affordability must transcend one-time subsidies to include systemic changes like income-based pricing models or public ownership of digital infrastructure for long-term sustainability. The term “rhizomatic,” rooted in its philosophical origins, aptly describes the interconnectedness and adaptability necessary for digital inclusion. By applying this lens, we can more effectively address the multidimensional barriers of the digital divide and design actionable, holistic solutions that align with the complexities of real-world challenges. Far from being an abstract concept, the rhizomatic framework serves as a practical guide for crafting policies and interventions that ensure digital health technologies are not only accessible but also equitable and inclusive, ultimately offering a transformative path toward sustainable digital inclusion.

In the context of digital health and technology, the rhizomatic framework highlights that no single component (e.g., access, affordability, availability, adequacy and acceptability) can be addressed in isolation without impacting the others. For example, availability—the physical infrastructure like broadband networks and cell towers—is essential but meaningless if the internet speeds (adequacy) are insufficient for telehealth or remote patient monitoring. Even if these technical requirements are met, cultural barriers, such as mistrust of digital platforms or linguistic mismatches (acceptability), may prevent individuals from engaging with the technology. Finally, affordability is a constant challenge, not just for initial access but also for staying current with rapidly evolving technologies like AI-based diagnostic tools or subscription-based telehealth services. The rhizomatic framework insists that these components are not standalone, rather they are interdependent and must be tackled together to truly eliminate the digital divide.

To illustrate the rhizomatic nature of digital health ecosystems, consider the rapid adoption of telehealth during the COVID-19 pandemic. While many patients gained initial access to telemedicine platforms due to relaxed regulations and temporary subsidies, the benefits were unevenly distributed. In rural areas, the lack of broadband infrastructure (availability) rendered telehealth services inaccessible for many. In low-income urban neighborhoods, even those with internet access faced challenges with inadequate speeds (adequacy) or devices not optimized for video consultations. Additionally, language barriers and a lack of culturally informed digital tools (acceptability) further excluded marginalized populations. As subsidies expired, affordability once again became a barrier, with patients unable to continue accessing these services. This example underscores the rhizomatic framework's core assertion: without addressing all components simultaneously, inequities persist, and piecemeal solutions fail to close the digital divide.

At the Media and Innovation Lab (MIL) at the University of Miami Miller School of Medicine, we have adopted the Rhizomatic Digital Ecosystem Framework to systematically address the digital divide and exclusion through targeted efforts in availability, adequacy, acceptability, and affordability. Our initiatives are designed not only to expand access but to ensure that digital inclusion leads to true equity, with measurable and impactful outcomes. To improve availability, MIL collaborates with partners to deploy advanced infrastructure solutions such as satellite-based internet systems as well as leveraging the potential of 5G wireless technology with its Fixed Wireless Access (FWA) capability, which extends high-speed internet to underserved and remote areas, bypassing traditional infrastructure barriers. These technologies significantly enhance the scalability and reliability of digital connectivity in hard-to-reach regions. Additionally, we have developed and distributed *The MIL Box*, a remote health monitoring solution designed to bridge the digital and healthcare divide. Approximately 1,500 MIL Boxes, equipped with six IoT health devices, a smartphone, and Wi-Fi connectivity, have been disseminated in urban and rural communities to address the multi-component aspects of the digital divide. Addressing adequacy, we ensure digital resources meet modern performance standards for activities like telehealth and education by monitoring bandwidth, device compatibility, and user satisfaction. Recognizing the importance of acceptability, MIL develops multilingual platforms and conducts digital literacy workshops tailored to address cultural and linguistic barriers, with measurable outcomes including literacy improvements, inclusivity assessments, and adoption rates. Affordability is tackled through advocacy for income-based pricing models and private-sector partnerships to subsidize devices and internet plans, with metrics evaluating cost as a percentage of household income, subsidy impacts, and disparities across demographic groups. To integrate these efforts, MIL employs a comprehensive Digital Equity Composite Index (DECI) that aggregates data across all components to provide a holistic evaluation of progress toward digital equity (30–32). This comprehensive Digital Equity Composite Index includes components similar to those in the Rhizomatic Digital Ecosystem Framework: Access, Affordability, Digital Skills, Device Availability, Online Services, Socioeconomic Factors, and Usage. These components assess and represent the overall state of digital equity in a specific area.

This approach emphasizes that digital inclusion, like healthcare, is a fundamental right, as the tools and connectivity required to participate in modern healthcare systems must be universally accessible. By ensuring accountability through data-driven evaluations, innovative solutions, and transformative programs like *The MIL Box*, MIL not only addresses immediate digital inequities but also provides a replicable and impactful model for achieving sustainable digital inclusion in diverse global contexts.

Our proposed framework seeks to move beyond simplistic definitions and approaches, incorporating a multi-dimensional lens that emphasizes the critical importance of affordability, availability, adequacy, and acceptability. The goal is not merely to bridge the divide but to completely dismantle it, enabling equitable access to the tools and opportunities necessary for meaningful digital participation in an increasingly connected world. However, as current policies stand, the divide persists, further entrenching systemic inequities in the digital age. Our newly proposed framework, *the rhizomatic digital ecosystem*, goes beyond single definitions which often centers around *access,* and includes a multi-component definition to include other equally important components such as *access, availability, adequacy*, *acceptability and affordability*.

### Access through rhizomatic digital ecosystem framework

Within the framework of the Rhizomatic Digital Ecosystem, access plays a dual role in relation to the digital divide, contrasting with the traditional Digital Divide and Exclusion Lens. In this context, access pertains to the capacity of individuals and communities to acquire and effectively utilize digital technologies and the internet. The traditional digital divide emphasizes the disparities between those who enjoy seamless access to technology and digital services and those who do not, a discrepancy often driven by socioeconomic factors, geographic isolation, or insufficient educational opportunities (19, 33, 34). Such exclusion can exacerbate existing inequalities, as individuals lacking adequate access may forfeit valuable educational resources, employment prospects, and essential services, which are increasingly migrating online. As society increasingly transitions to a digital-centric paradigm, it becomes imperative to comprehend and address these access gaps. Doing so is crucial for fostering inclusivity and ensuring that all individuals are empowered to participate fully in the digital age. The dual role of access is evident in two key aspects. Firstly, it pertains to the essential infrastructure required for individuals to connect to the Internet; in this sense, infrastructure serves as a crucial bridge that enables users to navigate the digital landscape. Without this infrastructure, individuals are unable to “cross the road” to engage with the World Wide Web. Secondly, the term “access” also refers to the availability of convenient opportunities for online engagement, such as the use of smartphones or access to computers in public spaces like libraries and community centers. However, it is important to note that while these public resources provide short-term solutions, they do not equate to the long-term benefits of having personal computer access at home.

Access to the internet is closely linked with its availability, as both dimensions assess the extent to which existing infrastructure can provide reliable connectivity. While these concepts are interconnected, they also serve distinct roles in evaluating who has Internet access and who does not. Access can be understood as the ability to utilize Internet services, which is contingent upon the availability of sufficient infrastructure. Without adequate investment in infrastructure, the concept of access becomes irrelevant. Additionally, the availability of services is inherently constrained by geographical considerations, including the presence of service providers in specific areas and the nature of the technological infrastructure that has been established. Consequently, access to the Internet can vary significantly based on location, highlighting the disparities that exist in connectivity across different communities.

Access to services is closely linked to both availability and adequacy, the latter of which evaluates whether these services meet contemporary needs, such as telehealth and remote education. Access serves as a critical connection to modern capabilities in a digital landscape, influencing how individuals interact with the world—for instance, through online banking, telehealth services, and remote learning. If these services do not meet established standards of quality and adequacy, the issue of access becomes irrelevant, as ineffective services fail to fulfill their intended purpose. Access to technology is closely linked to acceptability, which underscores the individual and cultural barriers that people face, such as language and gaps in digital literacy. Currently, there are various tools available to assist individuals who speak languages other than the primary language of their devices or who have disabilities, including hearing impairments. These tools are integrated into smartphones, laptops, and desktops, featuring functionalities like screen readers. Screen readers can read aloud text displayed on the screen, and users can customize the size, font, and color of the text, enhancing readability. Moreover, web browsers often include integrated translation services that can convert content from one language to another. Some websites provide additional features, such as the ability to change font styles or have text read aloud to users. While these technologies fall under the umbrella of accessibility, the responsibility for incorporating these features primarily lies with hardware manufacturers and software developers. However, there is currently no universal obligation for websites and applications to adopt accessibility standards, but standards are available for implementation in websites and applications by The W3C Web Accessibility Initiative (35). As a result, individuals with accessibility challenges may find themselves lacking necessary alternatives, further exacerbating existing barriers to access and acceptability (24).

Access to the Internet is closely linked to affordability, particularly in the context of financial barriers faced by low-income households. Many families and individuals struggle to meet the monthly costs associated with Internet service and the necessary devices, which may prevent them from attaining the access they require to benefit from online resources. When high-speed broadband services are prohibitively expensive, individuals and families may be unable to secure the connectivity necessary for educational, professional, and social engagement.

In recent years, some policies have emerged at the federal, state, and local levels aimed at alleviating the financial burden of Internet access for these vulnerable populations, as previously mentioned about the Affordable Connectivity Program (ACP), New York City Big Apple Connect Program, New York State internet law, Lifeline program, and Tennessee fiber-optic network municipal utility to provide high-speed internet access (11, 15–17, 36, 37). Consequently, the landscape of Internet access is undergoing significant transformation, raising critical concerns regarding the long-term sustainability and scalability of programs aimed at bridging the digital divide for low-income communities.

### Availability through rhizomatic digital ecosystem framework

Availability, within the digital divide and exclusion framework, focuses on the physical infrastructure required for internet access, such as fiber optic cables, cell towers, and broadband networks. Challenges often arise in rural communities, where infrastructure is inadequate or nonexistent. However, availability issues also manifest in urban and suburban areas. For example, in Seattle, homeowners in an urban neighborhood discovered their property lacked wired internet access despite neighboring houses being connected. Comcast, the only viable provider, quoted $27,000 to extend 181 ft of cable, forcing residents to rely on unreliable 4G hotspots. This demonstrates how availability interacts with other factors: affordability becomes critical when high costs restrict access, adequacy is compromised when 4G hotspots fail to meet modern needs, and acceptability is impacted when customers perceive the service as exploitative (34, 38, 39).

Addressing these challenges requires infrastructure investments and tailored solutions. Federal programs like the Universal Service Fund (USF) and Lifeline aim to improve availability and affordability by subsidizing infrastructure and providing discounts for low-income households. The E-Rate program focuses on adequacy, ensuring schools and libraries gain access to sufficient connectivity, while the Affordable Connectivity Program addresses both affordability and acceptability by helping consumers manage costs and service quality (40–43).

Despite these efforts, gaps remain. In 2022, Microsoft revealed that 120.4 million Americans lacked adequate internet speeds, far exceeding the FCC's estimate of 14.5 million (44, 45). This discrepancy underscores the limitations of traditional approaches. Urban areas face regional ISP monopolies, limiting competition and perpetuating inadequate services, while rural regions lack sufficient infrastructure. Even in urban apartment buildings, landlords' exclusive ISP agreements often leave tenants with no competitive options, highlighting the interplay of availability and access constraints (46–49). A rhizomatic approach to availability acknowledges these interdependencies and seeks holistic solutions (50–54).

The Rhizomatic Digital Ecosystem Framework redefines availability as part of an interconnected system, emphasizing its dynamic relationship with access, affordability, adequacy, and acceptability. Rather than viewing availability in isolation, this framework considers it a node within a broader network of interdependent factors. For instance, improving availability in rural areas using 5G Fixed Wireless Access (FWA) or satellite systems can simultaneously address access by providing foundational infrastructure, affordability by reducing deployment costs, and adequacy by meeting modern bandwidth demands (33, 38).

This rhizomatic approach enables solutions that traditional frameworks cannot achieve. For example, decentralized technologies like community-owned broadband networks bypass monopolistic ISP practices, ensuring availability while fostering affordability and acceptability. Municipal broadband initiatives, such as those in Chattanooga, Tennessee, demonstrate how interconnected solutions can address multiple challenges. Chattanooga's gigabit-speed municipal network not only enhanced availability but also reduced costs and improved adequacy, making it a model for inclusive digital access (15, 16).

Policy strategies under the rhizomatic framework are inherently adaptive and multifaceted. Breaking down ISP monopolies and promoting public-private partnerships are essential steps. By addressing affordability through subsidies and adequacy through technological innovation, these policies ripple across the ecosystem, enhancing availability and ensuring equitable outcomes. Such initiatives highlight the potential of rhizomatic strategies to create scalable, flexible models that adapt to diverse needs (34, 46).

Through this lens, availability becomes more than a static metric; it is a dynamic process influenced by and influencing other A's. For instance, increasing availability in rural Indigenous communities addresses adequacy by supporting telehealth and education, affordability through cost-effective technologies, and acceptability by tailoring solutions to local needs. Policymakers can use this framework to design holistic interventions that address the digital divide comprehensively, ensuring that infrastructure investments lead to sustainable, inclusive ecosystems.

## Adequacy through the rhizomatic digital ecosystem framework

Adequacy is another critical component of the digital divide and exclusion framework, which examines whether the available internet service meets modern needs, as connection quality and speed can vary dramatically between locations and service providers. Quality refers to having a reliable and stable internet connection as well as the ability to connect multiple devices simultaneously without slowing down or experiencing connection dropouts. Performance encompasses the home Wi-Fi network, the number of devices on the network, internet speed, and the types of websites accessed, such as streaming services or informational sites.

Internet speed is extremely variable across the United States. Speed refers to how fast a website loads or how long it takes to download or upload files. The FCC recommends that all internet service providers deliver broadband (Cable, Fiber Optics, Satellite, DSL) and mobile (LTE/4G/5G) speeds with a minimum download speed of 25 Mbps and an upload speed of 3 Mbps (55, 56). In densely populated urban areas, many ISPs boast superior broadband quality, with download speeds ranging from 200 Mbps to 1 Gbps and upload speeds of 10 Mbps to 940 Mbps. However, speed tests of local and in-home services often reveal contradictory realities, where these optimal download and upload speeds are seldom reached (52, 57–61). This discrepancy underscores the persistent challenges of achieving adequacy under the traditional framework.

Adequacy, as defined in the Rhizomatic Digital Ecosystem Framework, assesses whether internet services meet modern needs, such as sufficient speed and reliability for critical activities like telehealth, remote education, and other bandwidth-intensive applications. While traditional approaches to digital adequacy focus narrowly on technical benchmarks like upload and download speeds, the rhizomatic perspective expands this understanding to consider the dynamic and interconnected factors that influence the functionality and relevance of digital systems. This perspective emphasizes that adequacy is not a static concept but one that must adapt to evolving technological landscapes, social expectations, and individual user needs.

By redefining adequacy through a rhizomatic lens, policies and solutions are geared towards creating systems that can dynamically evolve rather than merely meeting static thresholds. In 2024 the FCC (Federal communication commission) released a benchmarks report (55, 62) on the recommended broadband speeds ISPs should follow from 25 Mbps/3 Mbps to 100 Mbps/20 Mbps. These recommendation from the report can be rolled back, slowing down progress. This approach inherently connects adequacy to other dimensions of the 5 A's framework, such as availability and affordability. For instance, ensuring adequate speeds in underserved areas often requires scalable infrastructure solutions—a need intrinsically tied to availability. Moreover, the cost of implementing these solutions intersects directly with affordability, as high-quality services must remain financially accessible to low-income households.

Solutions must also address systemic barriers in underserved urban neighborhoods, where inadequate speeds hinder meaningful digital participation. For these areas, targeted initiatives like localized broadband networks and community-driven technology solutions can be transformative. These localized efforts often depend on acceptability, requiring cultural and digital literacy considerations to ensure community buy-in and effective implementation.

The rhizomatic approach also calls for the inclusion of end-user feedback as a key metric for assessing digital adequacy. This feedback loop ensures that services align with real-world expectations and contexts, enabling solutions to address multiple “A's” simultaneously. For example, integrating user-driven insights could lead to broadband systems designed to accommodate diverse cultural needs (acceptability) while maintaining financial viability (affordability) and robust infrastructure (access and availability). Ultimately, this holistic understanding of adequacy fosters equitable and future-ready digital systems that empower all individuals to fully engage in a rapidly digitizing world.

### Acceptability through the rhizomatic digital ecosystem framework

Acceptability is a crucial component of the digital divide and exclusion framework, emphasizing societal and cultural factors that hinder meaningful engagement with technology. These factors include language barriers, technological hesitation, and limited digital skills. Central to this is digital literacy, defined by the American Library Association as “the ability to use information and communication technologies to find, evaluate, create, and communicate information, requiring both cognitive and technical skills” (63). Low digital literacy can result from limited access to devices, lack of exposure to technology, or disinterest, creating significant challenges in performing everyday tasks such as browsing the internet, shopping online, or using social media. Addressing these challenges requires integrating digital literacy with traditional education through comprehensive training programs to ensure that individuals are equipped to thrive in a digital world (64).

Technophobia, a persistent fear or aversion to using technology, further exacerbates digital exclusion. This condition often arises from a lack of exposure or confidence in navigating technological tools. Feelings of inadequacy and anxiety can result in avoidance behaviors, reinforcing perceptions of technology as complex or inaccessible. Factors such as upbringing, age, education, and socioeconomic status play significant roles in shaping an individual's relationship with technology. Solutions must therefore address these psychological and cultural barriers, fostering confidence and trust in technology to encourage engagement and mitigate exclusion (18, 64).

Within the Rhizomatic Digital Ecosystem Framework, acceptability is reconceptualized as a dynamic interaction between digital technologies and the cultural, social, and individual contexts in which they are deployed. Unlike traditional, linear approaches, this framework emphasizes decentralized and multidimensional efforts that adapt to the evolving needs of diverse communities. Acceptability transcends surface-level issues, such as language barriers or basic digital literacy, to focus on embedding technology into the cultural fabric of communities in ways that align with norms, values, and individual agency.

The Rhizomatic Digital Ecosystem Framework emphasizes that digital inclusion cannot be achieved by addressing individual components in isolation, as each dimension—access, availability, adequacy, affordability, and acceptability—is deeply interconnected. For instance, access provides the foundational infrastructure necessary for digital participation, yet without availability—ensuring that this infrastructure delivers reliable and culturally inclusive connectivity—marginalized communities remain excluded. Likewise, adequacy extends beyond mere connectivity, requiring that digital services meet cultural, linguistic, and functional needs, such as telehealth platforms adapted for diverse literacy levels. However, these efforts are often hindered by affordability, as many low-income users cannot afford internet access, let alone customized digital tools. Finally, acceptability shapes user adoption—if individuals perceive digital services as irrelevant or inaccessible due to language barriers, usability concerns, or distrust, even subsidized or widely available broadband remains underutilized. These interdependencies underscore why fragmented, single-issue policies fail to close the digital divide. Instead, comprehensive, community-driven solutions must integrate all five dimensions, ensuring that digital infrastructure, affordability programs, and technological design evolve together to promote equitable, meaningful, and sustainable digital inclusion.

Policies informed by the Rhizomatic framework prioritize participatory governance and co-design principles. Community leaders, educators, and local organizations must be involved in shaping digital tools that resonate with cultural values and lived experiences. For instance, participatory design processes can help build trust in historically marginalized communities by enabling direct input into technology development. Solutions such as culturally tailored digital literacy programs (65–67), which evolve iteratively with technological advancements, address both acceptability and adequacy. Such programs not only teach basic skills but also foster a sense of ownership and empowerment, increasing long-term engagement and trust.

Concrete examples include the development of mobile apps for indigenous languages or telehealth platforms designed for multilingual support (68). These tools can simultaneously address availability, adequacy, and acceptability by aligning with cultural practices while ensuring usability and functionality.

Evaluation metrics within the Rhizomatic framework extend beyond adoption rates to include qualitative indicators such as user satisfaction, cultural alignment, and participation in education, healthcare, and economic activities. For instance, community feedback mechanisms can assess whether digital tools meet the unique needs of diverse populations. Policymakers should mandate regular assessments of these metrics to ensure the continued relevance and inclusivity of digital platforms.

To address technophobia, interventions must provide foundational skills training, such as navigating operating systems or practicing online safety. Consistent exposure to technology through iterative learning processes can reduce anxiety and build confidence. Moreover, accessibility features, including screen readers, text-to-speech functionality, and multilingual interfaces, empower users with varying literacy levels and needs, fostering greater trust and engagement. These initiatives align with affordability by ensuring that solutions remain accessible to low-income populations. By embedding acceptability into a holistic, interconnected strategy, the Rhizomatic framework ensures that digital inclusion efforts address the root causes of exclusion while fostering trust, cultural relevance, and meaningful engagement. This adaptive, community-driven approach lays the foundation for equitable digital ecosystems that empower communities rather than marginalize them.

### Affordability through the rhizomatic digital ecosystem framework

The affordability of internet, broadband, and technology is a critical factor driving the digital divide. Within the digital divide and exclusion framework, affordability acknowledges that many individuals and families may be unable to pay for internet services, devices, and digital resources, even when these technologies are technically available and adequate for their needs. Research indicates that low-income households are significantly less likely to have access to the internet and digital technologies due to the high costs associated with these services. Affordability, as a component of the digital divide and exclusion, encompasses not only the cost of devices, software, and internet services but also the ongoing expenses for maintenance and upgrades. For many, particularly those from low-income households and marginalized communities, the financial burden of digital technologies and internet access creates significant barriers to entry. These barriers limit access to essential services like education, employment opportunities, healthcare, and social connectivity, thereby exacerbating societal disparities and inequalities.

A prominent challenge to affordability is the practice of digital redlining (39, 69), which poses systemic barriers to equitable internet access. The National Digital Inclusion Alliance defines digital redlining as “discrimination by internet service providers in the deployment, maintenance, or upgrade of infrastructure or delivery of services,” often resulting in disparities based on income, race, and ethnicity. This modern form of discrimination echoes historical practices like redlining during the Jim Crow era and other racially discriminatory policies in the United States. However, its impact now extends beyond African-American/Black communities, affecting all residents in underserved areas regardless of socioeconomic status, race, ethnicity, or religion. The practice perpetuates inequities by creating systemic obstacles to affordable, high-quality internet access. Addressing affordability as a critical component of the digital divide is essential to dismantling these financial barriers, thereby enabling equitable access to the tools and resources necessary for individuals and communities to thrive in an increasingly digital world (1).

Affordability challenges are compounded by high fees associated with broadband, internet, and digital technologies, which can be categorized into upfront, obvious, and hidden costs. While obvious costs include purchasing devices and ongoing service fees, hidden costs such as equipment maintenance, learning to use new technology, and mitigating cybersecurity risks often go unnoticed. Additional hidden fees, like rental charges, early termination fees, and data caps, disproportionately burden low-income households, creating further barriers to digital access. These financial challenges make it difficult for consumers to compare prices, manage unexpected expenses, and sustain consistent access to essential digital services. Without targeted policies to address these cumulative costs, efforts to bridge the digital divide will remain insufficient, leaving marginalized communities at a significant disadvantage.

These cumulative costs often create a significant barrier, preventing individuals and families from maintaining consistent and equitable access to digital tools and services. Furthermore, compounding expenses from bundled services can limit consumers' ability to make informed choices, perpetuating cycles of inequality and exclusion. Efforts to address affordability must therefore account for both direct and indirect financial barriers. Policies like income-based pricing models, government subsidies, and increased transparency in service fees can alleviate some of the burden. Additionally, enforcing stronger regulations against practices like digital redlining—where internet service providers limit infrastructure investment in low-income or minority-dominated areas—can help dismantle systemic inequalities in access and affordability. Addressing these structural barriers is essential to ensuring that digital inclusion is not just a possibility but a reality for all, regardless of socioeconomic status.

The Rhizomatic Digital Ecosystem Framework broadens the definition of affordability beyond the initial cost of internet access and devices, emphasizing its interconnected nature with other components such as availability, adequacy, and acceptability. Affordability within this framework considers the ongoing costs of maintaining access, upgrading technologies, and adapting to an increasingly digital society. For instance, in rural areas where traditional broadband infrastructure is lacking, residents often rely on expensive mobile data plans or suboptimal satellite internet services. A real-world example is the efforts of companies that provide satellite-based internet at reduced costs in underserved regions, providing a scalable solution for rural connectivity. However, these solutions alone do not address broader economic barriers. Public-private partnerships, such as the ConnectHomeUSA (67, 70) initiative, exemplify affordability solutions by providing subsidized internet access and devices to families in public housing, ensuring long-term digital inclusion. These initiatives demonstrate how affordability issues intersect with availability and require a holistic approach to create sustainable solutions.

Affordability also encompasses the hidden costs associated with modern digital participation, such as subscriptions to essential software, cloud storage, and AI-powered tools. For example, low-income students may struggle to afford proprietary software like Microsoft Office or Adobe Creative Suite, which are often essential for educational success. Open-source alternatives have proven to be effective low-cost options. Additionally, innovative financing models, such as Google's Chromebook Lending programs enable cost-effective device distribution. Governments and ISPs can also implement tiered internet plans, providing affordable entry-level options while accommodating households with higher usage needs. By addressing affordability through the lens of interconnected barriers—where availability, adequacy, and acceptability influence financial access—the Rhizomatic Digital Ecosystem Framework informs adaptive, innovative policies that promote long-term digital equity. Such an approach underscores the importance of sustained affordability measures, bridging financial gaps, and enabling full participation in the digital economy.

## Conclusion

Our manuscript underscores the urgent need to redefine the digital divide and exclusion beyond the traditional focus on access to internet, broadband, and devices. The digital divide is a complex and interconnected issue shaped by five critical dimensions: access, availability, adequacy, acceptability, and affordability. Each of these components plays a vital role in shaping individuals' ability to engage meaningfully with digital technologies, and failing to address them collectively perpetuates inequities across socioeconomic, geographic, and demographic lines. While ensuring basic access to digital tools remains fundamental, achieving true digital equity requires a nuanced, multidimensional approach that addresses the specific barriers and challenges each component presents.

The Rhizomatic Digital Ecosystem Framework provides a transformative lens through which we can better understand the intricate interplay of these dimensions and design tailored solutions to address them. As mentioned previously, addressing availability requires investments in innovative infrastructure solutions such as 5G technology and satellite-based internet systems for rural areas, while adequacy demands setting and enforcing minimum standards for internet speed and performance to meet modern needs like telehealth and remote education. Acceptability calls for culturally informed digital literacy programs and the incorporation of assistive technologies to ensure inclusivity, particularly for individuals facing linguistic, social, or technological barriers. Meanwhile, affordability, necessitates comprehensive measures such as income-based pricing models, transparent billing practices, and targeted subsidies to remove financial barriers for low-income households.

A critical insight of this work is the recognition that these dimensions interact differently across different contexts (e.g., rural, urban, and suburban). Tailored, community-specific interventions are essential to addressing the unique combinations of challenges that these diverse settings present. Furthermore, the digital divide does not exist in isolation but intersects with broader systemic inequities in healthcare, education, and economic opportunities. As healthcare increasingly integrates digital tools such as AI-driven diagnostics and telemedicine platforms, the digital divide risks becoming a major determinant of health disparities. Digital inclusion must, therefore, be viewed as a basic utility and a fundamental right, integral to ensuring equitable participation in a digitally driven society. This holistic redefinition of the digital divide calls for coordinated efforts from policymakers, private-sector stakeholders, and community organizations. By adopting a multidimensional approach grounded in the Rhizomatic Digital Ecosystem Framework, we can move beyond fragmented and superficial solutions to foster meaningful, sustainable digital inclusion. Recognizing and acting on these interconnected factors is essential not only for bridging the digital divide but also for promoting health equity, social justice, and economic empowerment in a rapidly evolving digital world. Through sustained commitment and innovative strategies, we can build a truly inclusive digital future that leaves no one behind.

## Data Availability

The original contributions presented in the study are included in the article/Supplementary Material, further inquiries can be directed to the corresponding author.
